# 

**Published:** 1894-08

**Authors:** 


### CONTENTS FOR AUGUST, 1894.

ORIGINAL ADDRESS. PAGE.

Address to the Graduates at the Eleventh Annual Commencement of Niagara Uuiversity Medical College. By HARLOW C. CURTISS, A. M. .... 1

ORIGINAL COMMUNICATIONS.

Diphtheria: How is it Propagated? By G. W. GOLER, M. D .... 9
Marasmus. By HERMAN D. MARCUS, M. D .... 15
Provision for Epileptics. By WILLIAM PRYOR LETCHWORTH, LL. D .... 18
Philadelphia Notes .... 30

SOCIETY PROCEEDINGS.

Medical Society of the County of Chautauqua .... 31

SELECTION.

The Dangers of the Long Rectal Tube .... 32

EDITORIAL.

State Licensure for the Practice of Medicine .... 33
Topics of the Month .... 35

Personal .... 39
Obituary .... 41
Society Meetings .... 41
Medical College Notes .... 44
Academy of Medicine Notes .... 46
Book Reviews .... 46
Literary Notes .... 60
Miscellany .... 62

